# A Case of Basal Cell Adenoma of the Upper Lip

**DOI:** 10.1155/2014/795356

**Published:** 2014-02-23

**Authors:** Masanori Kudoh, Hiroyuki Harada, Yuriko Sato, Ken Omura, Yoshimasa Ishii

**Affiliations:** ^1^Division of Oral and Maxillofacial Surgery, Ebina General Hospital, 1320 Kawaraguchi, Ebina, Kanagawa 243-0433, Japan; ^2^Oral and Maxillofacial Surgery, Department of Oral Restitution, Division of Oral Health Sciences, Graduate School, Tokyo Medical and Dental University, Japan

## Abstract

Basal cell adenoma is a rare type of benign salivary gland tumor found most commonly in the parotid gland. We present a rare case of basal cell adenoma arising in the minor salivary gland of the upper lip. The patient was a 59-year-old Japanese man who visited our department in December 2012 with a chief complaint of a mass in the upper lip, which had increased in size over several years. A mobile, elastic, and relatively soft mass without tenderness was palpable in the upper lip region. The mucosa of the upper lip covering the mass was normal. Tumor extirpation was performed under local anesthesia. Histologically, the tumor had a capsule and was composed of islands of relatively uniform, monotonous cells. Immunohistochemically, the inner tumor comprised tubuloductal structures that showed strong staining for CK7, while the outer tumor showed weak staining for CK7. The outer tumor cells also stained positively for CD10 and p63. The MIB-1 (Ki-67) labeling index was extremely low. Basal cell adenoma was diagnosed based on these results. The postoperative course was uneventful 12 months after surgery and there has been no recurrence.

## 1. Introduction

Basal cell adenoma (BCA) is a benign salivary gland tumor that most frequently arises in the parotid gland and is characterized by the basaloid appearance of the tumor cells and the absence of the myxochondroid stromal component present in pleomorphic adenoma. BCA accounts for 1–3% of all salivary gland tumors and is included as a separate salivary gland tumor in the 1991 World Health Organization classification [1]. Of 13749 primary epithelial salivary gland tumors, 160 cases of BCA were registered at the Armed Forces Institute of Pathology (AFIP) [2] in 1991. The main locations are the parotid gland (75%) and the minor salivary glands of the upper lip (20%) [3]. Here, we report a rare case of BCA arising in the minor salivary gland of the upper lip.

## 2. Case Report

**Figure 1:**
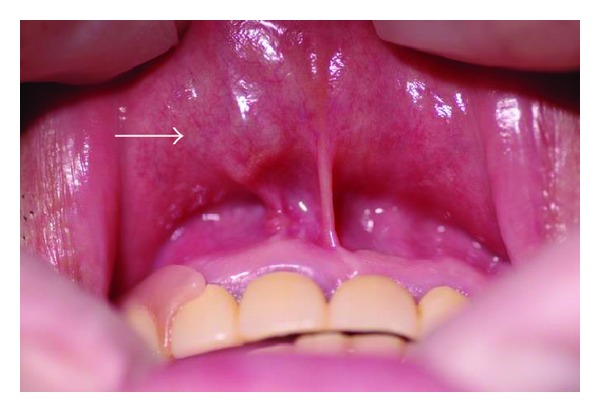
Intraoral view at the first visit. A mass measuring 1.0 × 1.0 cm was noted in the upper lip.

The patient was a 59-year-old Japanese man who visited the Division of Oral and Maxillofacial Surgery, Ebina General Hospital, in December 2012, with a chief complaint of a mass in the upper lip, which had increased in size over several years. A mobile, elastic, and relatively soft mass without tenderness was palpable in the upper lip. The mucosa in the upper lip covering the mass was normal. The patient had no relevant medical history. The mass in the upper lip region measured 1.0 × 1.0 cm (Figure 1). The clinical diagnosis was suspected to be pleomorphic adenoma arising in the minor salivary gland of the upper lip.

**Figure 2:**
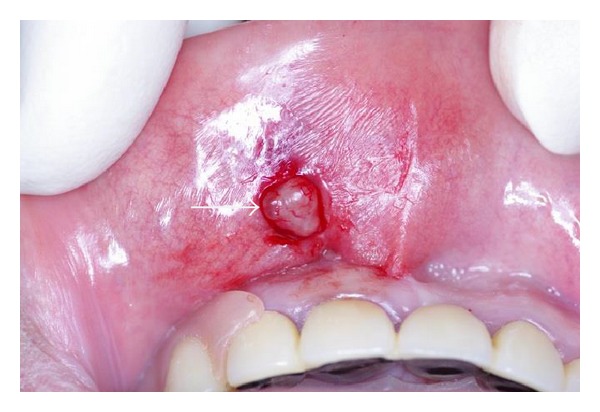
Intraoperative view. The mass had no adhesion and was excised with ease (arrow).

In December 2012, tumor extirpation was performed under local anesthesia. There was no adhesion to the surrounding tissue and the postoperative course was uneventful with no recurrence. Macroscopically, the surgical specimen consisted of an ovoid, nodular mass that measured 1.0 × 1.0 × 0.8 cm. The mass was solid, lobulated, and brownish-yellow in appearance (Figure 2). The tumor was surrounded by a thin capsule and did not involve any normal salivary gland tissue.

**Figure 3:**
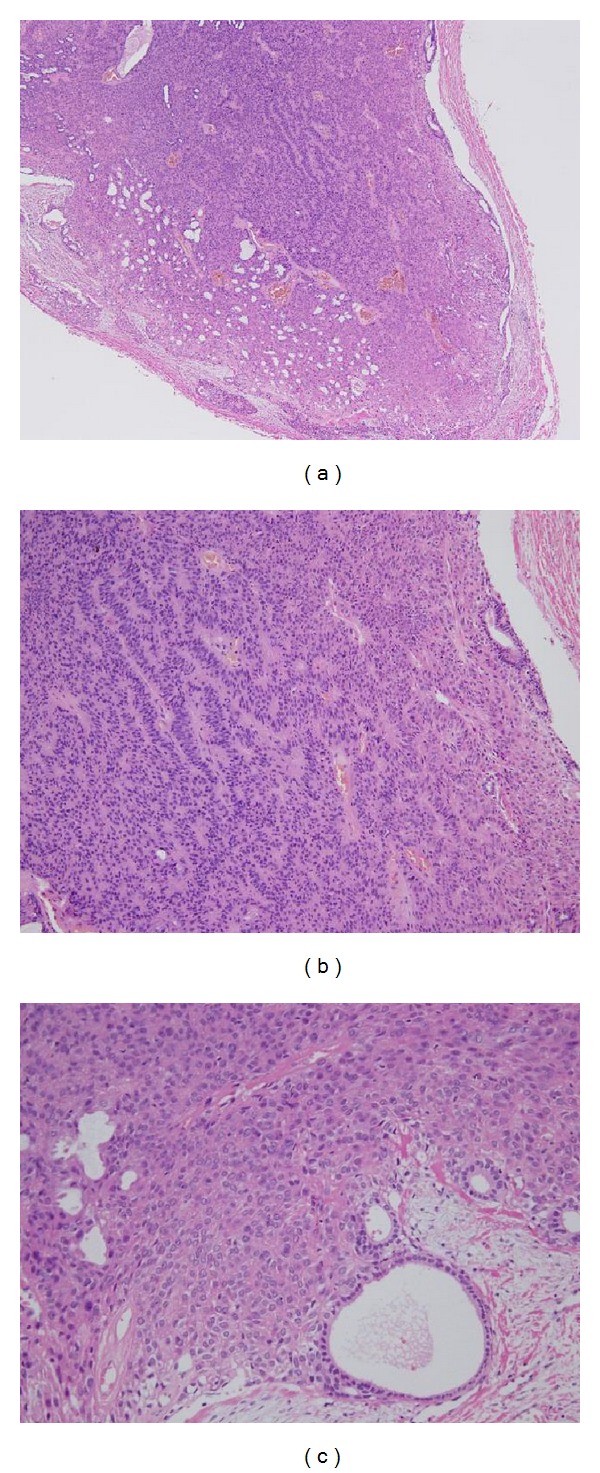
Histopathology of the surgical specimen using hematoxylin-eosin (H-E) staining. (a) The tumor was surrounded by a thin capsule and did not involve any normal salivary gland tissue (H-E ×40). (b) Proliferation of monomorphic epithelial cells with a trabecular pattern and variably sized nests of cuboidal cells (H-E ×100). (c) Proliferation of monomorphic epithelial cells of a tubular type (H-E ×100).

**Figure 4:**
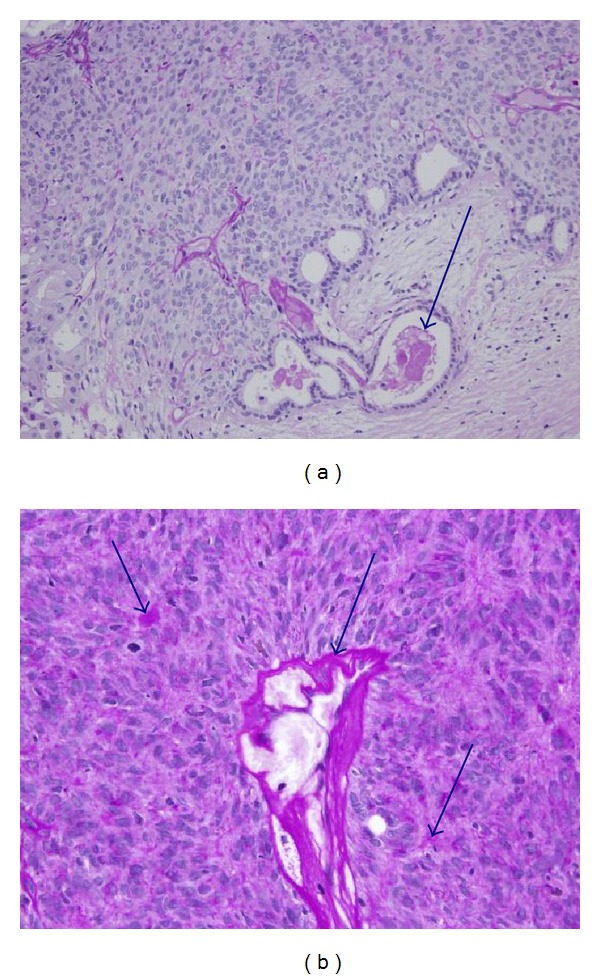
Histopathology of the surgical specimen using Periodic Acid-Schiff (PAS) staining. (a) A mucinous substance showing positive PAS staining was present in the lumen (arrow) (PAS ×100). (b) Dropwise PAS staining was found for a basement membrane-like material within and around alveolar nests in the form of a film (arrow). Deposition of abundant basal lamina material showing PAS staining was also observed (PAS ×200).

Histopathologically, the tumor was encapsulated by fibrous connective tissue and demarcated from the surrounding tissues (Figure 3(a)). It consisted of monomorphic epithelial cells with a trabecular or tubular pattern (Figures 3(b) and 3(c)). The solid nests were composed of almost uniform epithelial cells that were columnar or cuboidal in shape with scanty eosinophilic cytoplasm and round to ovoid nuclei. The stroma [4] surrounding the epithelial tumor nests was composed of thin fibrous tissue and was well demarcated from the solid nests (Figures 3(a) and 3(b)). Further analysis showed a glandular structure containing a mucinous substance that was positive in Periodic Acid-Schiff (PAS) staining (Figure 4(a)) and deposition of abundant PAS-positive basal lamina material within and around the tumor nests (Figure 4(b)).

**Figure 5:**
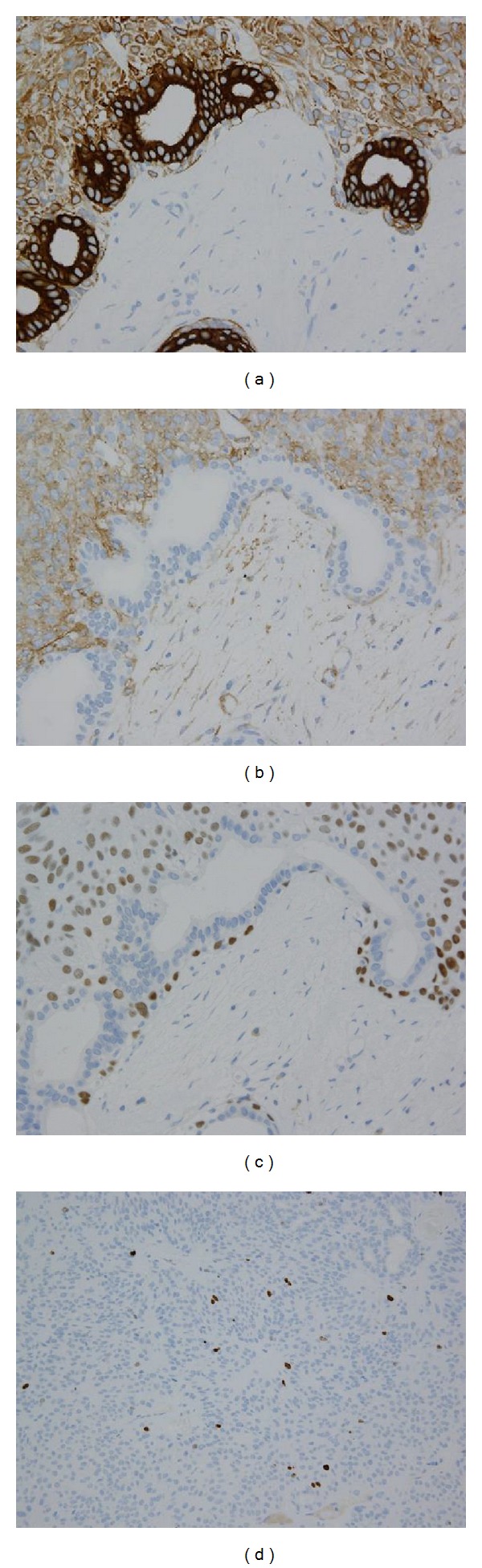
Results from immunohistochemistry. (a) CK7 (×100): lumen cells of glands showed strong staining and outer cells showed weak staining. (b) *α*-SMA (×200): outer cells showed cytoplasmic staining. (c) p63 (×200): outer cells showed nuclear staining. (d) MIB-1 (Ki-67) (×100): the MIB-1 (Ki-67) labeling index was extremely low.

Immunohistochemically, the inner tumor region of tubuloductal structures stained strongly positive for cytokeratin 7 (CK7) and the outer tumor cells were weakly positive for CK7 (Figure 5(a)). The outer tumor cells also showed cytoplasmic staining for *α*-smooth muscle actin (*α*-SMA) and CD10 and nuclear staining for p63 (Figures 5(b) and 5(c)). The MIB-1 (Ki-67) labeling index was extremely low (Figure 5(d)). These results led to a diagnosis of BCA.

## 3. Discussion

The incidence of BCA is low, accounting for 1 to 3% of all salivary gland tumors, with a peak in the seventh decade of life [5]. The main locations are the parotid gland (75%) and the minor salivary glands of the upper lip (20%) [2]. Fantasia and Neville have reported 50 cases of BCA arising in the upper lip in the minor salivary gland [6]. Mucoceles most commonly involve the lower lips of young patients (second and third decades), whereas the BCA typically occurs in older patients (mean age: 61 years) and usually involves the upper lip [6].

Histopathologically, BCAs are benign tumors composed of relatively isomorphic basaloid cells, a conspicuous basal cell layer, and distinctive basement membrane-like material. Most are well circumscribed and encapsulated, although a multinodular microscopic pattern may be found in any of the subtypes. BCAs lack the characteristic myxochondroid matrix found in pleomorphic adenoma [1]. BCAs are classified based on their morphologic pattern into four subtypes: solid, trabecular, tubular, and membranous [1], with the solid variant being the most common. The tumor in our case had a predominant tubular-trabecular pattern composed of islands of tumor cells with a hyperchromatic, palisaded, and peripheral layer of cells (Figure 3). The initial clinical diagnosis was suspected to be pleomorphic adenoma in the minor salivary gland in the upper lip. In such cases, immunohistochemistry is needed for differential diagnosis to exclude mucocele, malignant lymphoma, lymphangioma, hemangioma, adenocystic carcinoma (ACC), and basal cell adenocarcinoma (BCAC). Hiranuma et al. [5] also suggested that a mixed tumor should be considered in differential diagnosis for BCA. Important distinguishing features of BCAs include an absence of histologically recognizable myoepithelial cells and a sharp demarcation between the epithelium and stroma. The stroma in a BCA tends to be scanty and thereis an absence of the myxoid or chondroids elements that are present in pleomorphic adenoma.

Clinically, it is difficult to distinguish any asymptomatic tumor from BCA. Differential diagnosis of BCAC from BCA is also difficult because of the similar cellular composition of these two tumors [7]. However, low-grade carcinomas exhibit invasive, unencapsulated growth into adjacent soft tissue, often with associated perineural or vascular invasion.

BCA has sometimes been mistaken for ACC. Jung et al. [8] reported that, compared to BCA without capsular invasion, the BCACs and BCAs with capsular invasion were more likely to be larger and have solid or cribriform patterns and most BCACs and BCAs exhibited nuclear beta-catenin expression, and beta-catenin, CK5/6, CD117, and S-100 protein were helpful for differentiating basal cell neoplasms from ACC. BCAs with capsular invasion shared several pathological features with BCACs, including a large size and frequent cribriform patterns but the malignant potential of these tumors seems highly limited and should be reexamined. Jung et al. [8] also reported that beta-catenin immunostaining may aid the differential diagnosis between basal cell neoplasms and ACCs.

Therefore, we suggest use of the Ki-67 labeling index and beta-catenin immunostaining as one approach to distinguish BCA from BCAC.

Final diagnosis is difficult based on imaging and clinical findings alone. Thus, total excision and immunohistochemistry including Ki-67 labeling and beta-catenin should be performed to make a definite diagnosis. The recurrence rate is 25% for the membranous variant of BCA and malignant transformation of BCA has been reported [8]. Therefore, it is necessary to perform complete tumor excision. This approach was used in our case and the postoperative course was uneventful 12 months after surgery with no recurrence.
